# Wellness Assessment of Alzheimer’s Patients in an Instrumented Health-Care Facility

**DOI:** 10.3390/s19173658

**Published:** 2019-08-22

**Authors:** Andrea Masciadri, Sara Comai, Fabio Salice

**Affiliations:** Department of Electronics Information and Bioengineering, Politecnico di Milano, Via Anzani 42, 22100 Como, Italy

**Keywords:** quality of life, ambient assisted living, assistive technology, wellness assessment, indoor localization

## Abstract

Wellness assessment refers to the evaluation of physical, mental, and social well-being. This work explores the possibility of applying technological tools to assist clinicians and professionals to improve the quality of life of people through continuous monitoring of their wellness. The contribution of this paper is manifold: a coarse-grained localization system is responsible for monitoring and collecting data related to patients, while a novel wellness assessment methodology is proposed to extract quantitative indicators related to the well-being of patients from the collected data. The proposed system has been installed at “Il Paese Ritrovato", an innovative health-care facility for Alzheimer’s in Monza, Italy; first satisfactory results have been obtained, and the dataset shows great potential for several applications.

## 1. Introduction

The forecast of the United Nation [1] estimates that in 2030 the total number of elderly in the world will grow to 1.4 billion. Thanks to the growing longevity, and because of the declining fertility rate of the industrialized countries, this number is projected to reach an increment of 116% by 2050: the number of people aged more than 60 years old will be almost the double with respect to 2017, surpassing the number of adolescents and youth aged. This phenomenon is already impacting on the worldwide health-care systems that are facing with an increasing number of requests for assistance; for this reason a change in the current welfare paradigm adopted by the most advanced countries is necessary. Technology favours this change, promoting a preventive-care paradigm of welfare through monitoring tools that allow assessing the individual’s well-being and anticipating undesirable situations with corrective actions [2]. Keeping the patients healthy is now a key objective of the long term treatment centers: promoting the execution of specific daily activities it is possible to encourage the maintenance of physical and cognitive abilities, and to decelerate the progression of neuronal degeneration thanks to the development of a natural brain compensation.

### 1.1. State of the Art

The use of technologies to support patients and operators in residential facilities is a current goal of assistive technologies. Niemeijer et al. [3] evaluate the potential advantages, as well as the ethical implications, deriving from the application of surveillance technology in residential care. A first study has been proposed by Doshi et al. [4] who address the effectiveness of a localization system in supporting the activities of caregivers in a nursing facility, with promising results. We now present a brief literature review for indoor localization system and wellness assessment.

#### 1.1.1. Indoor Localization

Indoor localization has been widely addressed by the scientific community for several applications. This work focuses on RSSI (Received Signal Strength Indicator) based methodologies that can be used to estimate a human position in an indoor localization system; these methodologies can be divided into the following categories [5,6,7]: proximity detection (or winner takes all: a computational principle where the highest RSSI antenna is active, and responsible of the localization while all others are ignored), scene analysis (or fingerprinting), and trilateration. The proximity detection methods are the simplest in terms of implementation, attributing the target’s position to the strongest received signal [8]; the scene analysis techniques use scene details computed from a particular point of view to match patterns [9]; the trilateration techniques use the geometric properties to compute the target location [10].

The targeted use case requires low cost, high reliable, coarse grained localization system, where the key point is to guarantee the identification of the patient in an environment. Thus, the selected methodology for this work is based on proximity detection methodology.

#### 1.1.2. Wellness Assessment

Scientific literature does not contain a univocal definition of wellness: in the hedonic view wellness is identified as the absence of pain through the search of pleasure, while in the eudaimonic view wellness is the psycho-physical functioning of the individual. Ryan et al investigated this division in a survey on wellness [11]. Different terms have been identified in the literature: wellness, well-being or wellbeing, quality of life, and health-related quality of life are used to asses the status of health and life conditions of an individual, even though with slightly different meaning. According to Pinto et al. [12], comfort, well-being and quality of life have different interpretations, and their definition is subjective since it depends on the scope of the research; authors conclude that, even if this concepts share some attributes, researchers should not use them indistinctly. Comfort refers to the satisfaction of the individual needs; well-being refers to the spiritual and psychological spheres, while quality of life has a wider meaning and it is related to the individual perception of being well in general. Most surveys [11,13] conclude that researchers agree on the fact that wellness is a multidimensional construct; even the World Health Organization has defined wellness as “a state of complete physical, mental and social well-being and not merely the absence of disease or infirmity” [14]. Depending on the aim of the study, each model in the literature gives a different definition. According to Cella [15], the quality of life is a subjective and multidimensional concept including functional ability and physical, emotional, and social well-being. Felce et al. [16] consider quality of life as a multidimensional concept including development and physical, material, social, and emotional well-being. Another concept is the health related quality of life; according to Torrance [17] and Guyatt [18], this concept is strictly related to the physical sphere of the individual wellness. In conclusion, wellness is considered as a construct composed of multiple dimensions that are related to the psycho-physical health of the individual and to the interactions between the individual and the social environment. The most adopted wellness models are:

- Five factors model of personality [19] (or big five) defines five categories of human behaviour: neuroticism, extraversion, openness to experience, agreeableness, conscientiousness.
- The wheel of wellness model [20] defines five life tasks: spirituality, self-direction, work and leisure, friendship and love.
- The indivisible self model [21,22] defines five second-order factors that compose wellness: essential self, social self, creative self, physical self and coping self.

Measuring wellness. According to Roscoe [13], “several of these theories have been the foundation of instruments that measure wellness”; currently, professionals and doctors get a measure of a person’s well-being by filling in questionnaires. Among them, the most common are:

- Life assessment questionnaire (LAQ) [23] scores wellness with 100 questions on a five-point Likert scale. It provides a measure of the social, spiritual, physical, intellectual, emotional and occupational wellness of the individual.
- Perceived wellness survey (PWS) [24] scores wellness with 36 questions on a six-point Likert scale. It provides a measure of the social, emotional, physical, intellectual, spiritual and psychological wellness of the individual.
- Optimal living profile (OPL) [25] scores wellness with 135 questions on a five-point Likert scale. It provides a measure of the social, emotional, physical, intellectual, spiritual and environmental wellness of the individual.
- Wellness evaluation of life inventory (WEL) [26,27]. The latest version of this survey is the the WEL-S, it scores wellness with 120 questions on a five-point Likert scale. The 5F-WEL instead uses 91 items; among them, 17 are experimental items, on a five-point Likert scale. Finally, the 4F-WEL is an additional extension of the 5F-WEL that scores cognitive-emotional, relational, physical and spiritual wellness.
- Wellness inventory (WI) [28] scores wellness with 120 questions on a five-point Likert scale. It provides a measure of multiple dimensions such as self-responsibility and love, breathing, moving, sensing, thinking, eating, feeling, communication, playing and working, sex, finding meaning, and transcending.
- TestWell [29] scores wellness with 100 questions on a five-point Likert scale. It provides a measure of the social, emotional, physical, intellectual, spiritual and occupational wellness of the individual.

Table 1 reports the most adopted questionnaires (in the columns) together with the wellness dimensions that they consider (in the rows).

However, recent studies propose new ways to automatically assess wellness by analyzing the activities performed by a person; in particular, the aim is to measure the functional decline of patients monitoring the activities that people execute for self-care, such as sleeping, having lunch, toileting, etc. These kind of activities are known in the literature as activities of daily living (ADLs) [30,31], while the most common indicators are:

- The Barthel index [32] is currently used in hospitals to evaluate the self-care ability and mobility of patients. Its measure considers 10 basic ADLs to generate a score of independence. The environment greatly influence the final score.
- The Katz index [31] is used to assess the individual ability of performing ADLs independently. An overall performance on six basic ADLs is the result of the instrument.
- The MACTAR patient preference disability questionnaire [33] is used to assess the ability of the patients to perform five specific activities.
- The health assessment questionnaire [34] is used to assess the ability of patients to perform ADLs. It considers 20 items to describe eight basic ADLs.
- The modified health assessment questionnaire [35] is an alteration of the Health Assessment Questionnaire. It considers just 8 items (12 less than HAQ) to score the patient ability of performing ADLs.
- The PF-10 [36] is used to examine the physical ability of an individual through 10 items. It is a subset of the MOS 36-Item Short-Form Health Survey (SF-36): an instrument designed by the same authors to assess the health status of patients in a clinical settings.
- The functional independence measure [37] is used to estimate the level of autonomy in performing 18 basic ADLs. It is designed for adults who are independent in most functional activities.

### 1.2. Contributions and Paper Organization

This work aims to estimate the well-being of patients (mainly physical and social), giving to specialists and doctors a technological tool that supports their activities. The paper is organized as follows. Section 2.1 introduces the adopted Indoor Localization System that has been designed and validated in the mentioned use case; in particular, this section reports the system’s architecture, the localization algorithm, and the methodology that has been used to place sensors. Section 2.2 describes the indexes that have been designed for wellness assessment, distinguishing among physical activity indexes, social activity indexes, and psychological indexes. Section 2.3 refers to the system implementation, while Section 2.4 refers to the data collection phase. Section 3 reports the results that have been obtained, while Section 4 discusses the experimental results derived from the practical application of the introduced methods. Finally, Section 5 concludes the paper.

### 1.3. Case Study

Among the diseases that afflict the elderly and whose care requires huge investments, chronic degenerative diseases are of particular relevance. Dementia afflicts approximately 3% of people between 65 yo and 74 yo, 19% between 75 yo and 84 yo, and almost 50% of those over 85 yo [38]. Its symptoms involves the degeneration of cognitive abilities, creating memory, problem-solving, and language impairments that affect the regular and everyday activities of a person. Alzheimer’s disease is a particular form of progressively disabling degenerative dementia; unfortunately, despite the great number of studies that have been done to find possible treatments for Alzheimer’s disease, no definitive therapies have been found. For this reason, the management of patient’s needs becomes essential to increase their quality of life as long as possible.

The proposed system aims to provide a technological support to a health-care facility inhabited by people affected by Alzheimer’s disease; the evaluation of the system has been performed at “Il Paese Ritrovato” [39]: an innovative and pioneering health-care facility realized by La Meridiana cooperative  [40] in the city of Monza—Italy. The experimentation includes the implementation and the installation of a localization system that keeps the caregivers updated about the position and the condition of the patients, generating alerts whenever patients cross borders that are not allowed. This system has been designed to meet the requirements of this kind of facility; for example, the monitored patients are affected by a degenerative disease that limits their cognitive abilities, making their interaction with the system almost impossible (in the case of Alzheimer’s, in fact, the ability to learn is gradually canceled); for this reason, the localization system must not require any interaction by the users and must be as non-invasive as possible. Thanks to this system, useful data has been collected and analyzed to help doctors and professionals in assessing the individual’s well-being of patients. As it will be further discussed in the next paragraphs, this assessment involves the Physical activity, the Social activity and the Cognitive activity of the patient, and it constitutes the basis of behavioral drifts’ analysis [41]; changes in human behavior, such as changes in the attendance of environments or in the order of execution of daily activities could be automatically recognized by the system and reported to caregivers. A crucial aspect of this analysis is the collection of data that must be carried out in a non-invasive but constant way: a progression of the disease may lie behind a change in the patient’s daily routine.

## 2. Materials and Methods

**Figure 1:**
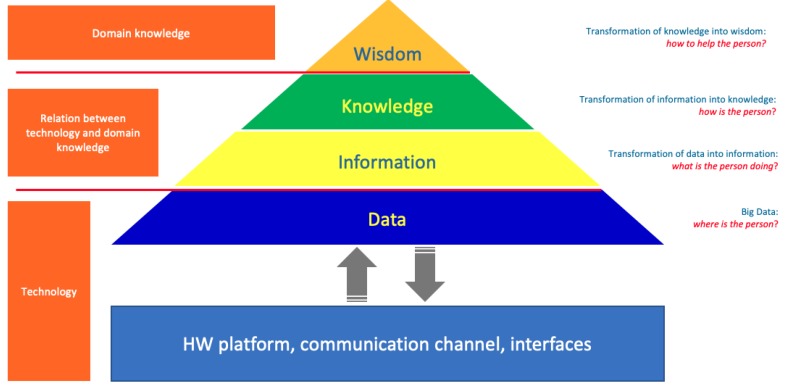
The applied methodology involves the transformation of the data collected by the sensors into knowledge that may be useful to support doctors in their activities.

The current welfare paradigm places prevention as the main objective to build a health-care system that can be considered efficient and sustainable. The work summarized in this paper proposes a method that involves ICT technology and defines a mechanism of synergistic cooperation among technical, social, and medical competences as a key factor to support health-care; Figure 1 shows the methodology that have been adopted, which refers to the transformation of data into wisdom. Thanks to a dedicated hardware platform, the system is capable to collect data related to the position of patients in the facility; this data are preprocessed and transmitted to a central server to be analyzed. Finally, in order to provide doctors with relevant information that can be part of their domain knowledge, it is necessary to group and process data in accordance with known wellness models.

This section provides the author’s contribution about coarse grained localization system (at the bottom of the pyramid) and wellness modeling (at the top of the pyramid). It is worth noting that the design of the entire system was made in such a way as to consider the patient’s well-being as an absolute priority; this question has also been debated for a long time in the literature [42]. The following section reports the outcomes of the experimentation: the localization system has been installed in “Il Paese Ritrovato", while the proposed wellness model has been validated using patients data; the experiment has been performed in collaboration with La Meridiana which collects the patients informed consent (including the agreement to use their data for scientific research).

### 2.1. Coarse Grained Localization System

The design phase of the proposed system has been driven by the requirements of facilities dedicated to the care of people with dementia and Alzheimer’s disease. The outcome is a coarse grained localization module based on iBeacon technology [43] constituted by disposable bracelets, fixed antennas, and a mobile application.

#### 2.1.1. Architecture

**Figure 2:**
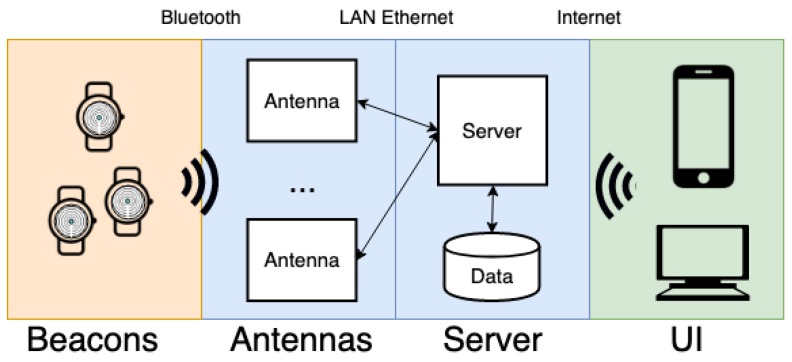
Architecture of the proposed indoor localization system.

Generally, traditional proximity detection systems place tags in fixed positions within the environment, and track smart devices that move in the space. On the contrary, the proposed system has been designed to reverse this approach: Bluetooth beacons (tags) are the mobile devices, while a set of smart antennas are installed in both internal and external environments. The proposed localization system is designed to operate in large structures with a consistent number of patients (in particular people affected by mild cognitive disabilities such as dementia) and caregivers. Due to specifications, the system has to be a coarse grained localization system meeting the necessary trade-off among costs, computational effort, and privacy. The localization system is RSSI based, with the proximity detection method and a set of constraints mechanism in order to avoid wall crossing. The system is composed of three main components (Figure 2): tags, antennas, and server.

At the time of their entry into the facility, each patient is assigned a tag, a beacon bracelet IP67 protection level, and he/she is aware of being localized while performing daily life activities. Beacons are bluetooth low energy (BLE) devices that broadcast packets with a standard payload (iBeacon advertising mode): a broadcasting power, a universal unique identifier (UUID), a major and a minor. The latter identify a specific device within a group. Transmitting a packet with a period of 100 ms (Apple iBeacon preset), a Beacon has an expected battery life up to 28 weeks; hence, a replacement of the batteries every six months is necessary.

The system is composed of several antennas that intercept the beacon messages broadcasted by the tags; the strength of the received packet—RSSI—is subsequently used by the system to estimate the position of tags. RSSI is strongly affected by noise like signal reflection, diffraction, and absorption. In particular, a human body behaves as an attenuator of Bluetooth signals, affecting signal strength and lowering accuracy; therefore, the position of the antennas is fundamental for a reasonable localization of patients. For this reason, particular attention was paid in the selection of the correct position of antennas.

The information transmitted by iBeacons in advertising mode, can be collected by a set of antennas [44] positioned in the countertop of rooms and common spaces, and in some keypoints in open spaces (e.g., garden lamps). The fundamental requirement of the selected device is that it has to have BLE modules, and an Ethernet/WiFi adapter for the connection with the server. Due to its technological characteristics, computational power, cost, market availability, certifications, and reliability, a Raspberry Pi3 [45] has been chosen to act as the antenna.

The software running on the antennas continuously performs a BLE scan to detect Bluetooth Beacons in the environment. Pre-processing is performed on the RSSI data received from each tag by applying a moving median filter. Each single sensor has been configured in order to have a specific filtering window; the window characterization is determined by the position of antenna and by the criticality of its role; in particular, increasing the length of the filter reduces the localization sensitivity, while increases the detection latency. For example, considering a transmission rate of 300 ms, the length of the filter has been set to fifteen samples for room antennas, and to sixty-five samples for outdoor antennas. It is worth noting that antennas are installed in strategic points so that the entire surface of the structure is covered. Furthermore, antennas are configured in room or corridor or external mode. The difference concerns the radius parameter, which determines the sensitivity of the antenna. Increasing the sensitivity of the antennas positioned in the corridor, for example, decreases the probability that the patient is mistakenly positioned in the room if his/her actual position is in the corridor; do note that some mistakes in the detected position have to be avoid for security reasons. The collected data (belonging to the detected iBeacons) are periodically transmitted to the server via an HTTP request.

**Figure 3:**
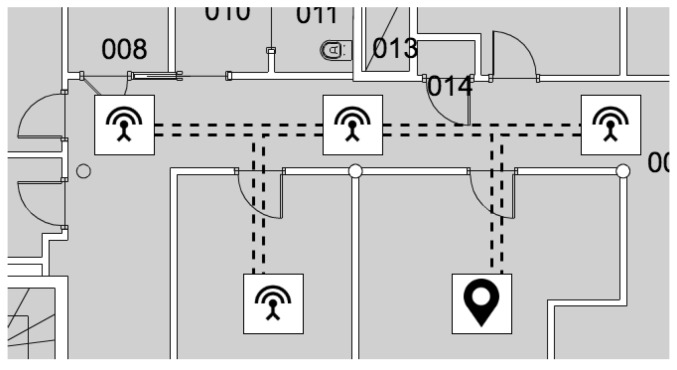
The map is modeled as an acyclic undirected graph where vertices (represented as squares) stand for antennas, and edges (represented as dashed lines) identify links between adjacent antennas. The black pin indicates the position of a patient that is always localized over a node.

The server is responsible for receiving and storing messages, and to compute the position of each patient according to the proposed proximity detection method. In order to avoid wall-crossing, the localization algorithm includes a constraint mechanism necessary to limit the admissible transitions between adjacent antennas; constraints has been modelled as an acyclic undirected graph (see Figure 3) G=(V,E) where the set of vertices *V* represents the antennas, while each edge in *E* defines the adjacency constraint between two antennas (two antennas are adjacent if and only if it exists a walkable path between them and there are no other antennas on the path). It is worth noting that antennas need to be correctly positioned in order to avoid any constraint violation; in particular, a transition point is needed when a person moves from one environment (i.e., room) to another one.

Two antennas are therefore said to be adjacent if a user can freely transit from one to the other as in Figure 3.

#### 2.1.2. Localization Algorithm

The presented localization system has been designed to support the work of professionals and doctors in an health-care facility dealing with people affected by Alzheimer’s disease. For this reason, the reliability of the system is particularly important and has been one of the most important driver for the designing phase. As mentioned in the previous section, the localization algorithm includes the implementation of a proximity detection method, partially adapted to avoid problems such as wall crossing. This method minimizes the computational effort, allowing a more reliable localization even in the case of noisy signals. It is worth considering that this methodology allows to a coarse-grained location (i.e., the person is always located under an antenna), unlike other methodologies which are computationally more complex (e.g., trilateration).


**Symbol**

**Description**

$TAG_{i}$
Bracelet of person *i*
$L_{i}\left(t\right)$
List of RSSI values of $TAG_{i}$ received from all antennas at time *t*
$CP_{i}\left(t\right)$
Candidate position of $TAG_{i}$ at time *t*
$SP_{i}\left(t\right)$
Selected position of $TAG_{i}$ at time *t*
$P(SP_{i}\left(t\right))$
Likelihood that the $TAG_{i}$ is in $SP_{i}$ at time *t*
$P_{entry}$
Initialization value of likelihood for each TAG
$P_{delta}$
Constant value added or removed to the likelihood
$P_{min}$
Likelihood threshold: under this level, wall crossing is allowed

The server is responsible for the computation of the position of all the tags. Considering a generic tag $TAG_{i}$ at time instant *t*, the list of messages $L_{i}\left(t\right)$ is analyzed. Elements with an RSSI value that is lower than a threshold $T_{min}$ are automatically removed from $L_{i}\left(t\right)$; the threshold is configured at installation time for every antenna, and it allows to adapt its sensitivity area with respect to the environmental characteristics: more sensible in wide areas and less sensible in small spaces (e.g.,  rooms).

Once the list of messages $L_{i}\left(t\right)$ has been computed, the proximity detection method is applied: the element with the highest RSSI value, is proposed as candidate position $CP_{i}\left(t\right)$ to locate the tag. The selected position of the tag $SP_{i}\left(t\right)$ is determined using the constraints to avoid wall crossing: $SP_{i}\left(t\right)$ is selected by evaluating the candidate position $CP_{i}\left(t\right)$ and the previous selected position $SP_{i}\left(t-1\right)$. The procedure is the following: if $CP_{i}\left(t\right)$ is equal to $SP_{i}\left(t-1\right)$, than $SP_{i}\left(t\right)=CP_{i}\left(t\right)$ and the likelihood that the $TAG_{i}$ is in $SP_{i}\left(t-1\right)$ rises of $P_{delta}$. On the contrary, that is $CP_{i}\left(t\right)\neq SP_{i}\left(t-1\right)$, the likelihood that the $TAG_{i}$ is in $SP_{i}\left(t-1\right)$ decreases of $P_{delta}$. If $P\left(SP_{i}\left(t\right)\right)<P_{min}$ then wall crossing is allowed, and the Selected Position $SP_{i}\left(t\right)$ is equal to the candidate position $CP_{i}\left(t\right)$. The pseudo-code is reported in Algorithm 1.

**Algorithm 1** Localization algorithm.
1:
**if** $SP_{i}\left(t-1\right)notset$ **then**
2:    //TAGi has not been localized before3:    $SP_{i}\left(t\right)\leftarrow CP_{i}\left(t\right)$4:    $P\left(SP_{i}\left(t\right)\right)\leftarrow P_{entry}$5:
**else**
6:    **if** $CP_{i}\left(t\right)=SP_{i}\left(t-1\right)$ **then**7:        //TAGi $didn^{\prime }t\;move\;from\;the\;last\;antenna$8:        $SP_{i}\left(t\right)\leftarrow CP_{i}\left(t\right)$9:        $P\left(SP_{i}\left(t\right)\right)\leftarrow P\left(SP_{i}\left(t-1\right)\right)+P_{delta}$10:    **if** $CP_{i}\left(t\right)\neq SP_{i}\left(t-1\right)$ **then**11:        **if** $adjacent(CP_{i}\left(t\right),SP_{i}\left(t-1\right))$ **then**12:           //TAGi moved to an adjacent antenna13:           $SP_{i}\left(t\right)\leftarrow CP_{i}\left(t\right)$14:           $P\left(SP_{i}\left(t\right)\right)\leftarrow P_{entry}$15:        **else**16:           //TAGi is constrained17:           **if** $P\left(SP_{i}\left(t-1\right)\right)>P_{min}$ **then**18:               //TAGi cannot jump19:               $SP_{i}\left(t\right)\leftarrow SP_{i}\left(t-1\right)$20:               $P\left(SP_{i}\left(t\right)\right)\leftarrow P\left(SP_{i}\left(t-1\right)\right)-P_{delta}$21:           **else**22:               //TAGi can jump23:               $SP_{i}\left(t\right)\leftarrow CP_{i}\left(t\right)$

24:               $P\left(SP_{i}\left(t\right)\right)\leftarrow P\left(SP_{i}\left(t-1\right)\right)+P_{delta}$


#### 2.1.3. System Notifications

The information about each patient’s position is visible to professionals and doctors via an application, mobile and web. In order to support the staff in managing security features, the system has been designed to anticipate, prevent and detect the escape of patients. The antennas positioned in proximity of the gates of the structure are configured to create a virtual fence with two security levels. Whenever a patient approaches the gates, he/she is detected by the antennas of the first virtual fence level and the system generates an alarm notification to all the operators of the structure. If the patient cross the gates, he/she is detected by the second virtual fence: this event generates an urgent notification for all the operators. Furthermore, a Kalman filter is currently under investigation in order to estimate patient trajectory and speed allowing a prediction of the escape. This information is used to generate a warning message before the first virtual fence in approached.

Another feature added to the system consists in detecting voluntary and accidental failures of the bracelet: hardware faults, battery problems, destructive actions perpetrated by the patients. In these cases, the wearable device does not transmit any signal: if the patient’s position is not retrieved for more than a minute a notification is generated to indicate to the operators a failure of the Tag, and its last position.

#### 2.1.4. Antenna Positioning

The localization system needs a strategy to find the best antenna positioning. Identifying the optimal number of antennas, and the relative position by taking into account the physical requirements of the environment, and the need to cover all the areas to be monitored, is a complex task. The antenna positioning algorithm has to minimize the implementation costs (hardware, installation, and maintenance), while a constraint about Relevant Areas must be guaranteed. About Relevant Areas, the position of antennas must be take into account the architecture of the environment and the role of each single room and space. For example, rooms where important activities take place require an appropriate concentration of antennas to detect patients and his/her movements. These essential areas (professionals and doctors determine the list of these areas according to their roles) are the most important areas in the indoor localization system. It is worth noting that incorrect localization of patients within essential areas is not acceptable.

Having said that, a method is proposed to define the various levels of priorities of antennas according to the activeness, importance, and functionality of the area covered by them. The definition of these priorities determines the order of antenna positioning: from high to low priority. Based on the characteristics and properties of the indoor and outdoor environment, and the functional requirement of the localization method (e.g., the necessity to have an intermediate localization point between adjacent rooms in order to avoid wall crossing), a scheme of three-level priority is proposed. The priority order of these three levels, from high to low, is: essential, auxiliary, and complementary. In case of areas with a particular aspect-ratio (e.g., a long and narrow area) or areas with a non regular geometry, areas are partitioned in “likely” squared blocks, and to each of them is associated an antenna. This operation has been named splitting.

**Figure 4:**
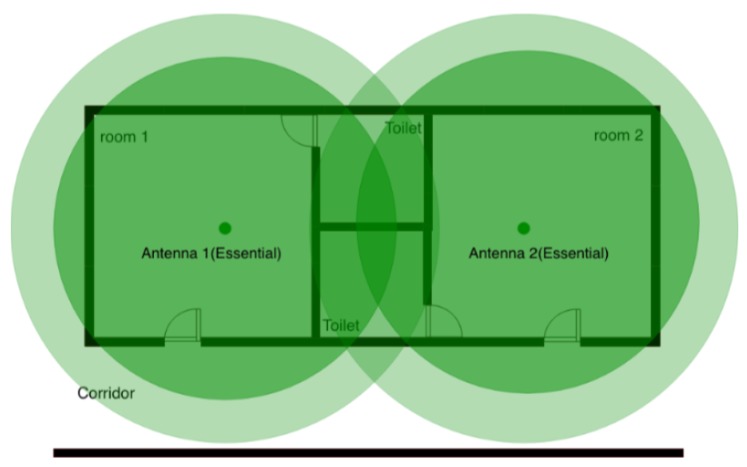
Simple positioning strategy for two essential antennas.

Essential antennas are placed to ensure the coverage of the essential areas. An area is defined as essential if the activity carried out in it is peculiar for the purpose of the health-care facility. For instance, in case of facilities where the autonomy is encouraged (as the proposed case study) bedrooms are essential points; in bedrooms patients are usually alone and out of caregivers’ sight for a long time. For the mutual tranquility of patients and caregivers the localization in these rooms is a relevant factor. Figure 4) shows how essential antennas could be placed to cover essential areas like bedrooms.

As previously described, the implemented localization method is proximity based with a mechanism of wall crossing avoidance. Since essential areas are typically not adjacent, a new set of nodes has to be added: the auxiliary antennas. Auxiliary nodes are fundamental for the implementation of the wall-crossing avoidance mechanism. For example, referring to Figure 4, when a person is in the bathroom he/she is in the intersection area of the two antennas located in rooms not physically accessible; in this case, the fluctuations of the Bluetooth signal is sufficient to make the person bounce back and forth between the two rooms. As also described in Section 2.1.2, in order to increase the reliability of the system, if antennas are not adjacent, the localization system freezes the patient waiting for an increased value of the likelihood. Auxiliary antennas represent the way to specify physical path among rooms having antennas that interfere without violate the physical constraints. Typically, an auxiliary antenna is placed when the sensitivity range of multiple essential antennas are overlapping each other and no physical connection between rooms exists; this occurs inevitably in most cases. It is worth noting that the sensitivity range of auxiliary antennas should be slightly overlapped with any other essential areas.

**Figure 5:**
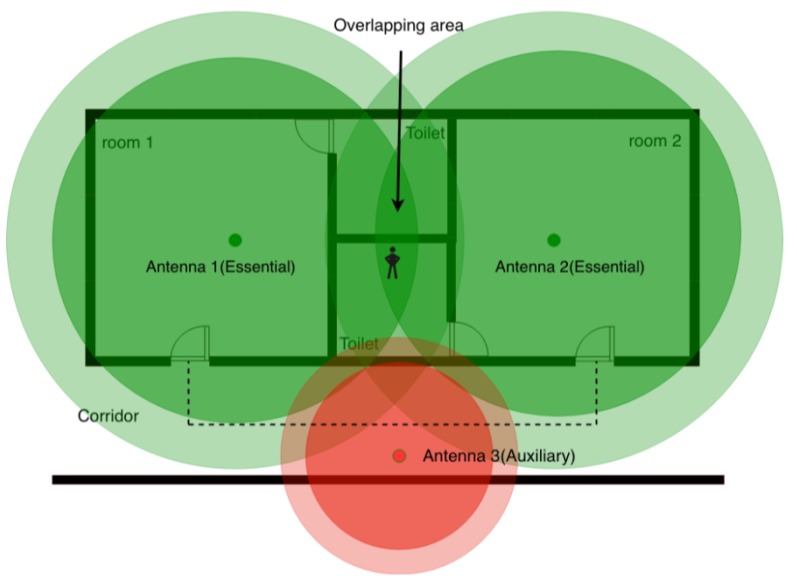
A scenario when an auxiliary antenna is placed to discriminate the essential areas. Placing an auxiliary antenna 3, possible localization errors are corrected. Anyone who walks from one room to another should pass through the corridor where the auxiliary antenna is placed.

Figure 5 shows the function of auxiliary antennas intuitively. If an inhabitant stays in the toilet (the overlapping area) of one bedroom, a localization error can easily happen: a person could pass from one room to another. The approach consist in placing a new auxiliary antenna on the corridor; thus, the jump from one room to another room is hindered.

Complementary antennas aim to locate inhabitants in indoor/outdoor environments (general areas) that are not considered neither essential areas nor transition areas among essential areas. Complementary antennas can even be omitted when the number of antennas is limited (e.g., for budget limitations). The main difference between essential areas and general areas is whether erroneous localization of inhabitants is acceptable or not when they stay in such areas. Unlike essential areas, in general areas the accuracy of the localization result is not a priority.

Due to unusual geometries with respect to those covered by an antenna (i.e., almost circular sensitivity area) there could be blind or overhung zones. These phenomena are circumscribed applying a splitting policy whose role is to cover these special areas with likely squared surfaces. In general, these antennas are named splitting antennas and are placed in the center of these subareas.

**Figure 6:**
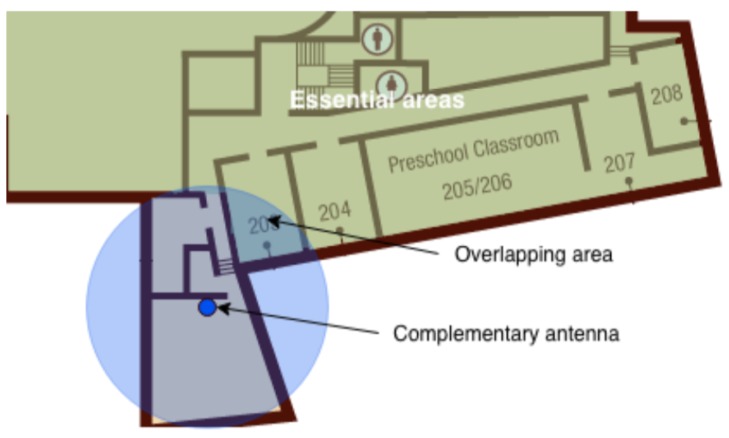
A scenario where the split of complementary antennas is needed.

**Figure 7:**
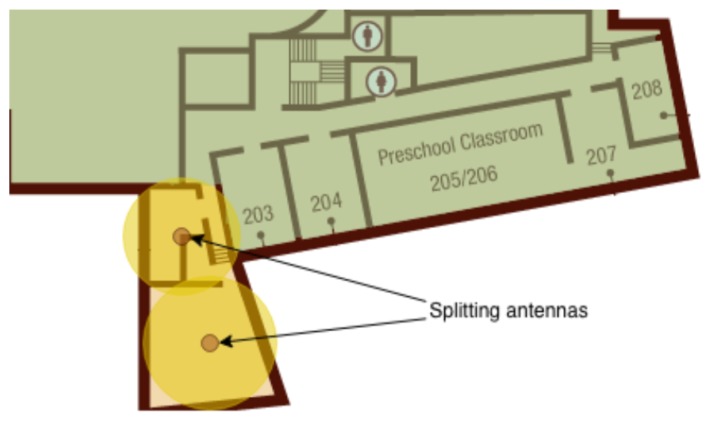
A scenario of splitting: after splitting, the overlapping area is reduced.

Figure 6 indicates a typical example where the splitting antennas are needed. Then, Figure 7 shows that the complementary area is split in two subareas; in this way the overlapping area is reduced.

In summary, essential and auxiliary antennas ensure precise localization of the inhabitants in high priority areas, while complementary antennas are used to cover the general areas that do not require high accuracy.

### 2.2. Wellness Assessment

This section aims to identify some tools for assessing a person’s well-being through technological support. In particular, attention was focused on the use of localization data (i.e., the position of the person) collected through a generic monitoring system to cover the physical and the social dimensions of the individual’s wellness. The following indicators, developed on the basis of the proposed models, can be constantly calculated and analyzed by technological supports, providing additional information for framing an individual’s state of well-being.

The reason behind this study is the simplicity with which localization data can be collected, and the potential benefit to use a continuous monitoring system that can improve the individual’s well-being. In the context of health-care facilities such as rest homes, where few operators are in charge of monitoring and assisting multiple patients, more and more facilities are being equipped with localization systems to monitor the position of patients. Thus, implementing the following indexes constitutes an important step for the long-term monitoring of the health status of the patients, without resorting to the installation of additional hardware infrastructures and without burdening the work of the operators.

Based on the data available from the localization system presented in the previous paragraphs, we now present a list of indicators that can support professionals and doctors in assessing the patient’s well-being. In relation to the aforementioned wellness models, we have separated the indicators into three categories: physical activity, social activity, and psychological activity.

#### 2.2.1. Physical Activity

Assessing the physical well-being of an individual is fundamental. Indeed, it is well known that practicing regular physical exercises is very useful for the prevention of many diseases; on the other hand, physical inactivity and sedentary behaviour are well-known risk behaviours, which may lead to unpleasant health outcomes such as strokes, heart disease, obesity, diabetes, hypertension, depression, high blood cholesterol, and anxiety.

Movement index provides an indication of the physical dimension of the individual; it computes an estimation of the quality and quantity of the movements that a patient performs during a day.

**Figure 8:**
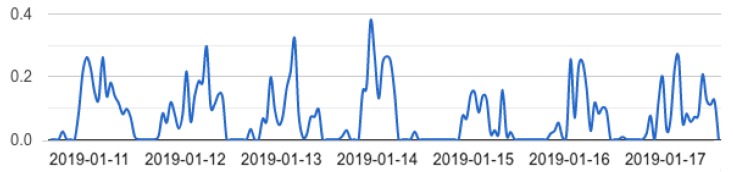
Movement index graph. This index computes an estimation of the physical activity of a patient during the day. The ordinate axis indicates the index value (between 0 and 1), while the abscissa axis constitutes the observation time.

Given the localization data, an estimation of the meters covered by the monitored subject are computed every day. Assigning a target value (i.e., the number of meters a patient is expected to travel) to each patient, it is possible to compute the movement index; thus, this index takes values between 0 (little movement) and 1 (enough movement)—Figure 8.

An extension of this index considers a meteorological variable in the normalization formula; practically, the algorithm penalizes a sedentary life-style on days with a favourable climate (sun, mild temperature etc.), while accepting less movement on adverse days.

Sleep index provides another key aspect of the physical dimension of an individual. Indeed, the National Institute for Health [46] states that insomnia can increase the risk for mental health problems as well as overall health concerns. For this reason, Reed et al. [47] presented a sleep efficiency index as the composition of multiple parameters collected during the patients’ sleep. These parameters are the sleep onset latency (SOL), the total sleep time (TST), the wake after sleep onset (WASO), the time attempting to sleep after the final awakening (TASAFA), and the duration of the sleep episode (DSE), where the DSE is computed as follows:

(1) DSE=SOL+TST+WASO+TASAFA

The sleep efficiency (SE) [47] is defined as:

(2) $$\mathrm{SE}=\frac{\mathrm{TST}}{\mathrm{DSE}}.$$

This index, as proposed by Reed et al. [47], provides a precise estimation of the sleep efficiency of patient, but it requires a careful observation of the patient’s activity to distinguish the different parameters. Considering the cost of the sensors required to measure all of this parameters, a simplification of this formula is introduced; this formula can be computed using only the localization data provided by the proposed localization system. Thus, the contribution of SOL, TASAFA, and TST must be cancelled since there are no tools to measure when a patient is awake in bed.

#### 2.2.2. Social Activity

As for the physical activity, even the Social Activity is a very important component of an individual’s well-being as it allows us to feel less lonely, angry, or disconnected. Promoting a safe and enjoyable environment, encouraging interactions with others, and building positive emotions are essential steps to build our social well-being. According to the taxonomy proposed by Levasseur et al. [48], social activities can be divided in: being with others, interacting with others, and participating to common activities. While the detection of the interaction among people requires dedicated devices, the other social activities can be inferred using localization data.

In the following indexes the period *T* is equal to the number of seconds in a day on which the metrics are evaluated. For example, considering all the day, *T* is equal to 86,400.

Isolation index (IsI) gives an indication of the social dimension of individual wellness. Where applied, it provides effective monitoring tools to assess social well-being in primary social networks (e.g., family, friends, relatives, etc.), and proximity networks (e.g., neighbours, shopkeepers, etc.).

To compute this index, the system should integrate information related to several people; indeed, for every person, the algorithm computes a score according to the number of people localized in his/her proximity. This index is computed once per day at midnight; first of all, for each monitored person *i* a vector $P_{i}=\left[P_{i}\left(1\right)P_{i}\left(2\right)\ldots P_{i}\left(n\right)\right]$ is computed. Each element of the vector $P_{i}\left(t\right)$ denotes the ID of the antenna corresponding to the position of the person at instant *t*. Furthermore, a second vector $S_{i}$ of length 86,400 is computed. Initially, this vector is initialized to zero; then, each element of the vector $S_{i}\left(t\right)$ is set to one whenever other people are located in the surrounding area occupied by person *i* at time *t*. Finally, the Isolation Index for person *i* is computed as follows:

(3) $$IsI_{i}=1-\frac{\sum_{t=1}^{T}S_{i}\left(t\right)}{T}.$$

The isolation index takes values between 0 (the monitored person has never been close to other people in that day) and 1 (the monitored person has been close to other people all the day).

Independence index (IdI) provides an estimate of the average tendency of a person to be in the company of doctors, caregivers and operators. For this reason, the Independence index helps doctors to evaluate the autonomy level of a patient; furthermore, this index is useful to those who administer health-care facilities because it provides an indication of the workload to which operators are subjected in the various work shifts. The vector $S_{i}$ is computed as explained for the isolation index but considering only the position of operators, doctors and professionals. Thus, the independence index for person *i* is computed as it follows:

(4) $$IdI_{i}=\frac{\sum_{t=1}^{T}S_{i}\left(t\right)}{T}.$$

The independence index takes values between 0 (the monitored person has never been close to a professional/doctor in that day) and 1 (the monitored person has been close to a professional/doctor all the day).

Relational index (RI) aims to estimate the interrelation between two persons quantifying the amount of time that they spend together in a day. For each couple of monitored persons *i*, *j*, the corresponding vectors of positions $P_{i}$, $P_{j}$ are computed as for the isolation index. Furthermore, a vector $V_{i,j}$ of length 86,400 is computed. Initially, this vector is initialized to zero; then, each element of the vector $V_{i,j}\left(t\right)$ is set to one whenever both person *i* and person *j* were occupying the same place at time *t*. Finally, the Relational Index for persons *i* and *j* is computed as follows:

(5) $$RI_{i,j}=\frac{\sum_{t=1}^{T}V_{i,j}\left(t\right)}{T}.$$

The relational index takes values between 0 (the two monitored persons have never been close to each other in that day) and 1 (the two monitored persons have been close to each other all the day).

**Figure 9:**
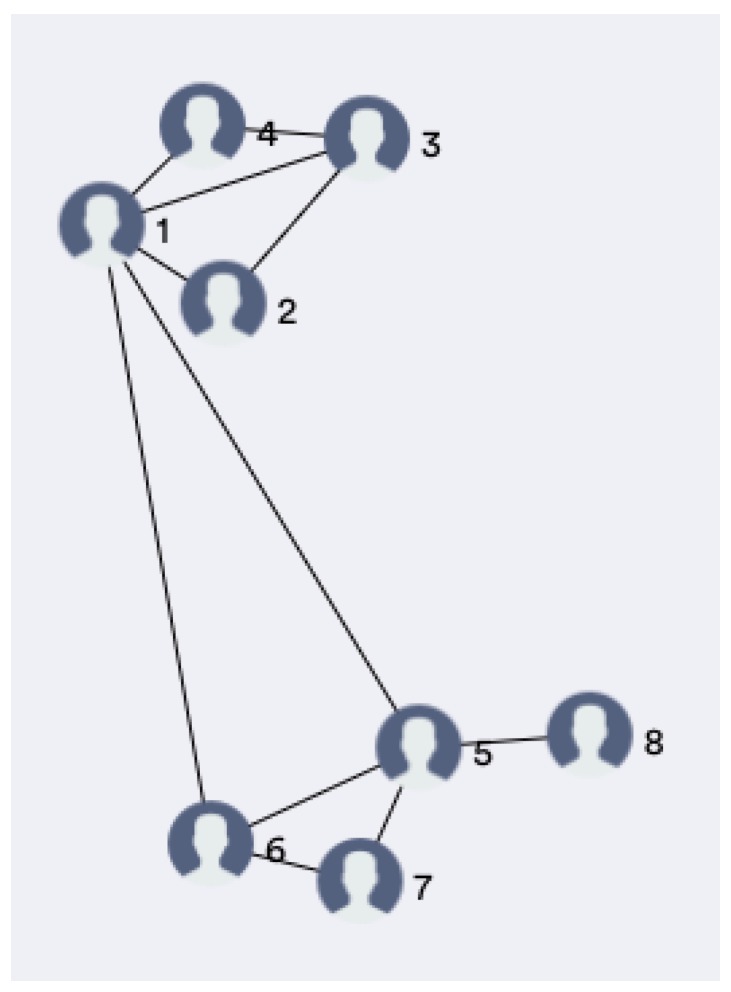
Nodes in the graph represent people, while arches represent relationships. The shorter an arc is, the greater the Relational index between the connected people.

The evaluation of this index among people in a community allows creating a graph of the personal relationship. An example is provided in Figure 9, where people are represented by nodes, and relationships are represented with arches: the length of an arch is proportional to the relational index between the two persons.

#### 2.2.3. Psychological Activity

**Figure 10:**
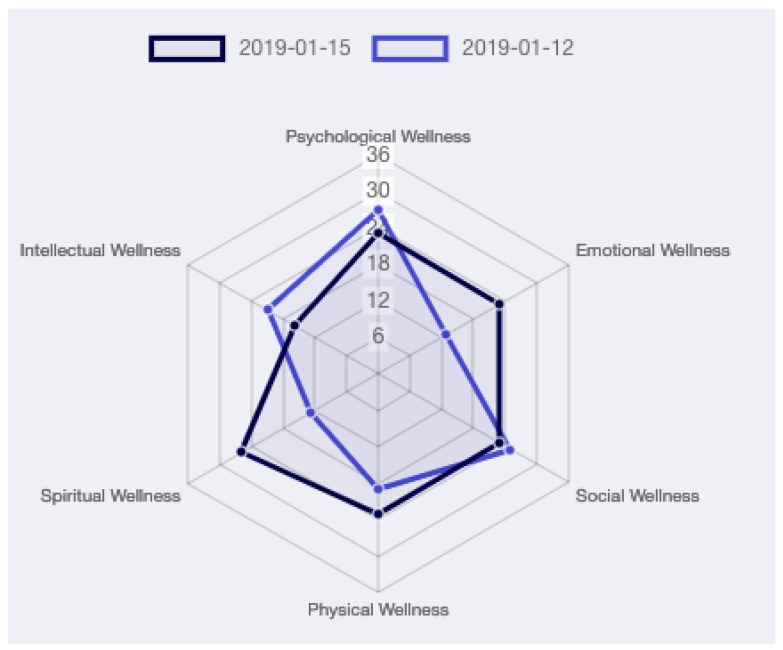
PWS graph. The technology provides doctors with tools for collecting, displaying and analyzing well-being indicators over time. Considering the multidimensional nature of wellness, the radar chart is excellent for displaying the trend over time of all the considered dimensions.

The wellness models proposed in Section 1.1.2 agree that in order to obtain an overall assessment of an individual’s status, all its dimensions must be taken into account. Although the discussion of the psychological domain is outside the scope of this work, it is worth to stress how much it is connected to health [49]; indeed, Grossi et al. [50] speaking of positive technology, support the importance that technology can have in improving people’s quality of life. For this reason, the proposed system includes a tool to support the activity of doctors and clinicians in evaluating the psychological activity of patients in an health-care facility. This tool allows visualizing questionnaire in a graphical form, allowing professionals to evaluate the evolution of each dimension of the individual’s wellness over time. Figure 10, for example, shows the outcome of the PWS questionnaire.

### 2.3. System Implementation

The localization system has been implemented according to the design choices reported in Section 2.1: the antennas positioning method guarantees the coverage of the entire surface of the structure, while the localization algorithm guarantees good performance in detecting the movements of people. The testing phase of the proposed solution was done thanks to the participation of the structure’s staff. For the entire duration of the experimentation periodic meetings were held with the aim of receiving a direct feedback to improve the initial idea. The meetings involved gathering information via questionnaires to all those involved in the project, from operators to facility managers, allowing satisfactory results to be achieved from all points of view.

### 2.4. Data Collection

A preliminary data collection phase has been performed tracking the localization of 54 patients for a period of almost 5 months at “Il Paese Ritrovato". This procedure was carried out taking into account the European directives regarding the GDPR, through informed consent managed directly by the managers of the structure; moreover, all data have been stored and processed only after making them anonymous.

**Figure 11:**
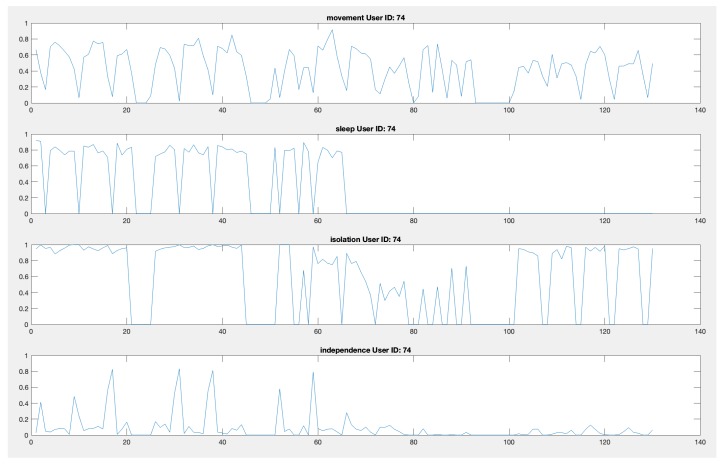
Movement index, sleep index, isolation index, and independence index for a single patient over the 130 days of data collection. The graphs show qualitatively the presence of cyclicity and anomalies (missing data) in the indicators.

The collected data have been subsequently analyzed to retrieve daily wellness indicators for every patient of the facility. Figure 11 shows the movement index, the sleep index, the isolation index, and the independence index for a single patient over the 130 days of data collection. Indexes take values between 0 and 1 as reported in Section 2.2. Doctors and professionals of “Il Paese Ritrovato" can view this chart for weekly, monthly or six-monthly periods to assess patients’ well-being.

## 3. Results

A new ICT methodology for wellness assessment has been presented; the main objective of this study is to acquire information on the position of patients in a health-care facility, to aggregate and elaborate them and to make them available to medical personnel to support their activities. For this reason, we have designed, installed and tested a localization system for the acquisition of the data that were then processed to constitute the well-being indicators presented in the previous paragraphs. The system is constituted by 107 essential antennas (i.e., rooms, kitchen, common spaces, etc.), 32 auxiliary antennas (i.e., corridors), and 14 splitting antennas.

Our case-study is “Il Paese Ritrovato", a self-contained community where patients are free to move and to interact with each other in wide open spaces, and where structures have been specifically designed to meet the patient’s needs, freeing them to the burden of feeling slaves of the disease. The residence consists of eight apartments with eight private rooms each. Each apartment has some common rooms such as the kitchen, the dining room and two living areas. A square is located in the center of the village, where residents can socialize visiting special buildings dedicated to them: a bar, a hairdresser, a cinema and a church. La Meridiana has thought a new concept of residence, where everything is designed to support the lives of Alzheimer’s patients. Thanks to the collaboration of many research institutions, the structure is constantly looking for innovation with cutting-edge technological systems, environmental and service design. In order to reduce costs while preserving the independence of the patients, only one professional (also called operator) is in charge of following a group of eight patients: one professional for each apartment. At their first entry into the facility, patients and operators are given a TAG bracelet that allows their location. The proposed system meets the residence requirements since it provides a low-cost technological tool to assist caregivers, professionals, and doctors in monitoring patients and accessing their wellness. Indeed, as it was required by the facility, few operators can monitor all the patients located in the rooms and gardens of the residence, just with the help of a smartphone. Finally, this system does not require patients to interact with it, feature that makes it very suitable to monitor people affected by Alzheimer disease.

Although we cannot yet make a quantitative analysis on the collected data (because of the still limited amount of data that we have collected), some considerations can be made on the evidence drawn during the evaluation phase. Figure 11 highlights possible issues and potentiality of the solution. For example, zero values that are shared among all the indexes denote periods of missing localization; this may be caused by the patient leaving the facility or by technical problems. As can be observed in particular in the sleep index chart, the dataset is quite incomplete, in this case due to a problem with the antenna in the room. Possible problems during the acquisition period include: problems of power supply of the antennas, loss of antenna connection, discharge of the battery of the bracelets, which must be monitored and checked.

**Figure 12:**
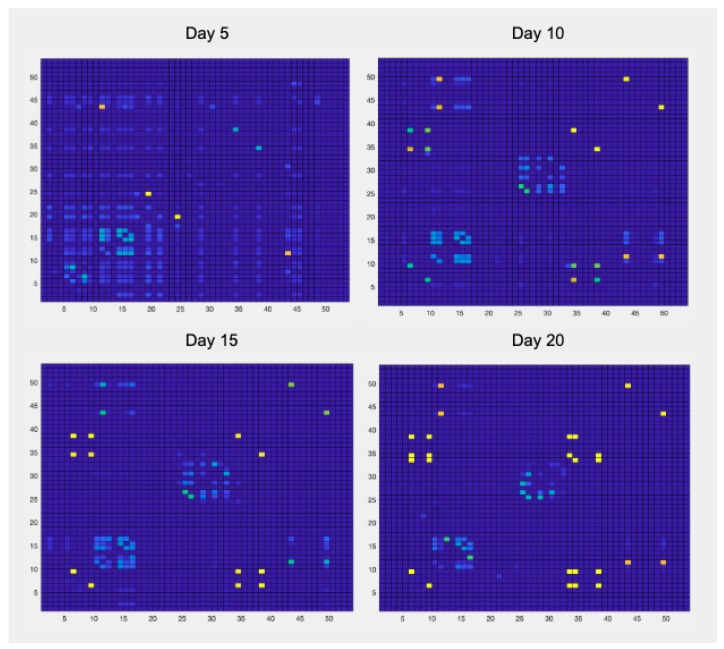
The relational index matrix shows the relational index for every couple of patients within the facility; values on the diagonal are not to be considered. The matrix shows the level of the relationship existing between each pair of patients in the structure (for this reason the matrix is symmetrical). High index values were represented by yellow color, low values are represented by blue color.

Finally, as it has been previously described in Section 2.2.2, the collected dataset also provides an indication of the interpersonal social relations. Figure 12 shows the evolution of the relational index of the patients evaluated in four time instants every 5 days. This type of visualization is made available to the staff of “Il Paese Ritrovato" to have an indication of the relationships that are being formed between couples or groups of patients. Higher values of the relational index are colored in yellow and indicates a frequent interaction between the related patients, while lower values are colored in blue. This matrix is reported for four different time instants, every five days: it is possible to notice, in a purely qualitative manner, groups of connected people and to highligt changes over different periods of time.

The experimentation conducted in the nursing home entailed the interaction of doctors, caregivers and patients with a localization system based on technological devices. The level of technology acceptance of patients and caregivers has been very positive. In general, patients did not reject the product, but from the analysis of the dataset and consulting with the health-care staff it emerged that for some patients using wearable devices is critical: in fact, they sometimes refuse to wear the TAG because they do not recognize it as their own. This topic is currently being examined by the professionals of the facility who are evaluating alternative TAGs to increase the level of confidence between patients and the object.

## 4. Discussion

The experimentation confirmed what emerged also from the literature analysis: assistive technologies are a valid tool to support patients and caregivers. During the last meeting, operators rated the system as very useful for supporting their tasks: considering the dimension of the structure, the unconventional freedom of the patients and the limited amount of human resources, the activities of the caregivers have been significantly simplified thanks to the information generated about patients’ movements. Furthermore, the generation of a dataset related to the movements of multiple Alzheimer’s patients is of fundamental importance to allow new research related to activity recognition, behavioral drift detection, as well as the identification of some characteristic phenomena for PWD such as wandering and sundown syndrome. As future work we plan to strengthen the data acquisition system by integrating new environmental sensors to monitor daily life activities and social relations between the patients of the facility.

## 5. Conclusions

A system for wellness assessment has been proposed; in particular, it allows to evaluate physical and social well-being of Alzheimer patients living autonomously in a protected health-care facility. The first contribution of the paper is the implementation of a coarse-grained low-cost localization system for continuous monitoring of the position of people wearing Bluetooth bracelets. While designing the system, particular attention has been given to reliability issues and to define a methodology for antennas positioning. The second contribution of this paper is the introduction of new quantitative indicators related to the well-being of patients that are computed from the collected data. These metrics, derived from the Quality of Life literature, provide doctors with relevant information that can be part of their domain knowledge. The proposed system has been installed at “Il Paese Ritrovato”, a highly innovative health-care facility for people affected by Alzheimer’s disease in Monza, Italy. The experimentation shows the potential of the proposed solution in mitigating the stress and anxiety suffered by caregivers, improving clinical knowledge on the disease, enhancing safety and promoting the patients’ autonomy, all outcomes that are particularly relevant for the future of dementia care.

## Figures and Tables

**Table 1:** Questionnaires to assess individual’s wellbeing.

	*LAQ*	*PWS*	*OLP*	*5F-WEL*	*4F-WEL*	*TestWel*
*REFERENCE*	[23]	[24]	[25]	[27]	[27]	[29]
*social*	✔	✔	✔	✔	✔	✔
*emotional*	✔	✔	✔		✔	✔
*physical*	✔	✔	✔	✔	✔	✔
*intellectual*	✔	✔	✔			✔
*spiritual*	✔	✔	✔		✔	✔
*occupational*	✔					✔
*psychological*		✔				
*environmental*			✔			
*creative*				✔		
*coping*				✔		
*essential*				✔		
